# Characterization of the two-dimensional length and diameter distributions of gold nanorods by size exclusion chromatography

**DOI:** 10.1038/s41598-025-90941-0

**Published:** 2025-03-12

**Authors:** Lukas Hartmann, Nabi Traoré, Wolfgang Peukert, Johannes Walter

**Affiliations:** 1 Institute of Particle Technology (LFG), Friedrich-Alexander-Universität Erlangen-Nürnberg (FAU), Cauerstraße 4, 91058 Erlangen, Germany; 2 Interdisciplinary Center for Functional Particle Systems (FPS), Friedrich-Alexander-Universität Erlangen-Nürnberg (FAU), Haberstraße 9a, 91058 Erlangen, Germany

**Keywords:** Colloids, Optical back coupling, Plasmonic gold nanorods, Size exclusion chromatography, 2-Dimensional characterization, Techniques and instrumentation, Characterization and analytical techniques

## Abstract

Access to complex multidimensional property distributions of nanoparticle systems is indispensable for the understanding of their synthesis, processing and application in modern production technologies. Plasmonic gold nanorods are a system of particular interest due to their shape-dependent localized surface plasmon resonance. In this study, we show how the optical back coupling technique, previously developed for the analysis of sedimentation coefficient-resolved extinction spectra derived from analytical ultracentrifugation experiments, can be transferred to standard laboratory equipment, namely size exclusion chromatography. The optical back coupling method utilizes the unique spectral extinction of plasmonic nanoparticles such as gold nanorods and other geometries combined with their hydrodynamic properties to determine full size and shape distributions. Our technique opens up a simple and easy-to-use characterization platform that requires very little sample volume and provides multidimensional access to length, diameter, aspect ratio, volume and surface area distributions of plasmonic nanoparticles in one single experiment. We characterize a variety of gold nanorods of different aspect ratios and validate our results by complementary scanning transmission electron microscopy experiments. Finally, we provide an outlook on how this approach can be developed further.

## Introduction

In the last decades, plasmonic gold nanoparticles (AuNPs) emerged as a revolutionary class of material with profound implications for various applications in research and technology, ranging from electronics^1,2^ and catalysis^3–5^ to medicine^6–9^ and energy conversion^10–12^. The properties of NPs and thus their potential applications largely depend on their size, shape, surface, composition, structure and their respective distributions^13^. Thus, the complex nature of NPs demands a comprehensive understanding of their disperse properties which necessitates multidimensional characterization techniques.

The unique size- and shape-dependent properties of plasmonic gold nanorods (AuNRs) make them attractive for various scientific and technological applications. Their tunable surface plasmon resonance within the visible (Vis) and infrared (IR) range and thus their ability to absorb and convert near-infrared (NIR) light into heat has enabled their use in photothermal therapy for cancer treatment demonstrating effective photothermal ablation of cancer cells using AuNRs in combination with NIR laser irradiation^14^. Furthermore, Kim et al. demonstrated the use of AuNRs as ultrasensitive surface-enhanced Raman spectroscopy substrates for the detection of trace amounts of analytes^15^ and Choi et al. reported the use of AuNRs as electrocatalysts for the oxygen reduction reaction in fuel cells^16^, showcasing their potential to enhance energy conversion efficiency.

In order to optimize the properties of AuNRs and to meet specific application requirements, an in-depth understanding of the NPs’ size and shape distributions is mandatory. Over the last decades, a variety of analytical techniques emerged to characterize size and shape distributions of NPs: As imaging techniques, transmission electron microscopy (TEM) and scanning electron microscopy (SEM) offer high spatial resolution and allow precise morphological analysis with respect to size and shape distributions but require expensive equipment and careful sample preparation. To obtain statistical relevant particle size distributions (PSDs) and shape distributions, a large number of NPs needs to be counted which makes TEM and SEM characterizations time-consuming. UV/Vis spectroscopy is quick as well as non-destructive and provides information on the AuNRs’ optical properties and plasmon resonances. However, it only gives averaged data for the entire sample, so information on the polydispersity is lost. Dynamic light scattering (DLS) provides hydrodynamic size and size distribution information rapidly but can be affected by aggregation and has limited resolution for polydisperse distributions as well as shape anisotropy^17,18^. Small-angle x-ray scattering (SAXS) can also provide information about size and shape distributions of AuNRs^19^, but requires, similar as all other ensemble based techniques including UV/Vis, DLS and SAXS, the deconvolution of the ill-posed underlying Fredholm integrals, to obtain the PSD. Hence, a combination of techniques is often necessary to gain comprehensive insights into AuNR characteristics.

A particularly strong technique, which can give multidimensional access to complex particle systems such as AuNRs is analytical ultracentrifugation (AUC)^20^. AUC provides high-resolution information on PSDs but is limited to 1-dimensional (1D) information when only sedimentation properties can be analyzed. This is the case for AuNRs as diffusion properties are typically not accessible due to their large mass. To overcome this obstacle, we developed the optical back coupling (OBC) technique in our group for the evaluation of sedimentation coefficient data derived by AUC with multiwavelength extinction detection^21^. Due to the characteristic plasmonic shift as a function of size and aspect ratio, a combined analysis of sedimentation coefficients and related extinction spectra gives rise to a second dimension and full 2-dimensional (2D) distributions of size and shape can be determined by a single AUC experiment. Recently, we transferred the OBC method to other plasmonic materials such as gold bipyramids^22^ and spherical gold-silver alloy nanoparticles^23^.

However, while this technique offers excellent resolution and provides statistical meaningful data, its applicability is so far limited as AUC requires considerable investment and know-how for operation. To extend the applicability of OBC for the determination of 2D shape distributions of AuNRs and other anisotropic NPs, we herein aimed to transfer our OBC method to high-performance liquid chromatography (HPLC) as a well-accessible separation and characterization technique in most laboratories. While the majority of chromatographic separation modes rely on specific interactions of individual compounds with the stationary phase, size exclusion chromatography (SEC) separates molecules or NPs by their hydrodynamic size within the mobile phase^24–26^. For anisotropic particles such as AuNRs, the particle’s hydrodynamic size can be linked to its length and diameter. For this, different hydrodynamic tools such as bead modelling^27,28^, the direct calculation using boundary element method^29,30^, or the path integration technique^31–33^ can be used, which give rise to the translational friction coefficient among other hydrodynamic properties. As SEC is a relative method, a calibration for the determination of molecular weights or PSDs is mandatory. By measuring the retention volume of multiple calibration standards, a calibration curve can be constructed to obtain a direct relation of molecular weight/hydrodynamic diameter with retention volume. From this calibration curve, the distributions of unknown samples can be determined^34^.

In this study, we demonstrate first that AuNRs can be separated very well by SEC based on differences in the hydrodynamic diameter under the assumption of sufficiently repulsive interactions between the AuNRs and the stationary phase material. Second, we show how the method of OBC can be transferred to SEC allowing the fast and precise 2D characterization of AuNRs by standard laboratory equipment. We test our method with a variety of AuNRs of different length and diameter distributions and validate the obtained 2D PSDs by complementary scanning transmission electron microscopy (STEM) analysis.

## Theoretical considerations

### Size-exclusion chromatography

SEC emerged as the standard technique for the separation and determination of molecular weight distributions of polymers. Recently, we expanded the applicability of SEC to the classification and characterization of spherical NPs^34^. In SEC, molecules or NPs are separated according to their hydrodynamic size within the surrounding mobile phase. The dispersed NPs are pumped through the chromatographic column which is packed with porous stationary phase particles. While small NPs can diffuse into most of the available pore volume, larger NPs have only access to the larger pores. Thus, their residence time is shorter compared to small NPs and the overall retention volume decreases with larger hydrodynamic diameters. From the SEC separation mechanism, it is obvious that the separation takes place in the pore space only and that the separation range is solely influenced by the pore size distribution of the stationary phase.

**Fig. 1:**
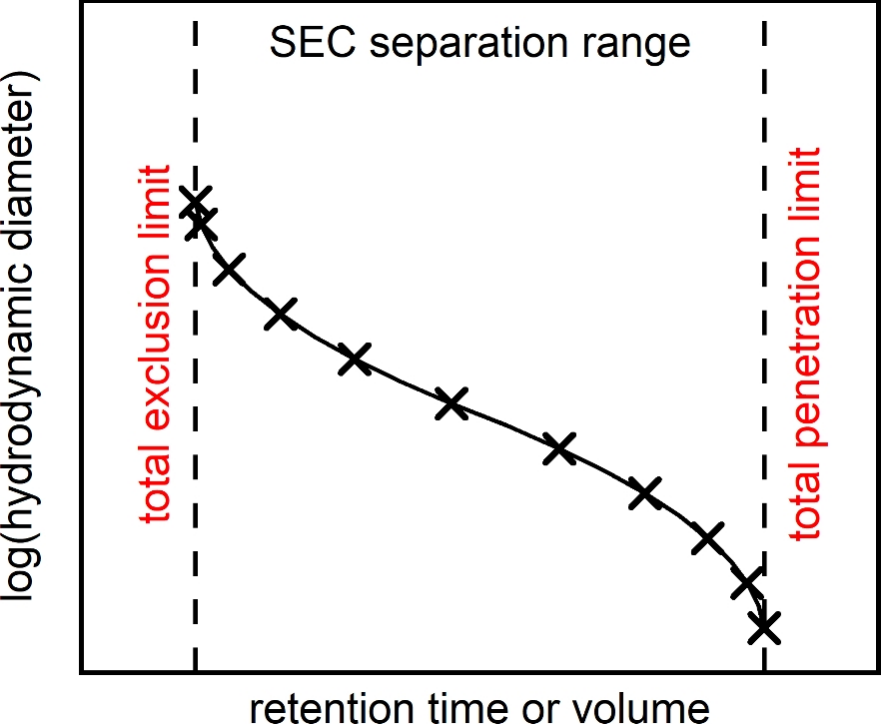
Schematic representation of the SEC separation range and calibration curve. The dotted lines indicate the total exclusion and penetration limit defining the separation range of the column.

As depicted in Fig. 1, the whole separation range is limited by the total exclusion limit and total penetration limit. NPs larger than the largest pore size are completely excluded from the pore space and elute at the total exclusion limit of the column preventing classification by SEC. The total penetration limit describes the retention of NPs smaller than the smallest accessible pore size. Here, separation by SEC is also impossible since all NPs can diffuse into the whole pore volume and thus elute at the same retention time. The calibration curve is constructed by sequentially injecting standards of known size into the column. The respective diameters are then plotted against the measured retention time or volume as shown by the crosses in Fig. 1. By fitting a (usually) polynomial function of 3rd or 5th order or by using a spline interpolation as done here, the calibration curve is obtained.

In chromatography, refractive index (RI), multi-angle light scattering (MALS) and many other detectors are frequently used. In case of AuNRs, diode array detectors (DADs) are advantageous due to their capability to capture the pronounced size- and shape-dependent surface plasmon resonance of the AuNRs in the Vis and NIR range. DADs offer the possibility to gain access to the complete optical responses of eluting AuNPs providing not only valuable information on the retention characteristic but also on size, shape, composition and potential agglomerates within the initial PSD^35–38^. Typically, full extinction spectra ranging from the UV and Vis up to the NIR with acquisition rates of several Hz can be extracted. Thus for each retention volume, full information on the optical properties are accessible for further data analysis such as the herein proposed OBC approach.

### Optical back coupling

#### Framework and implementation

**Fig. 2:**
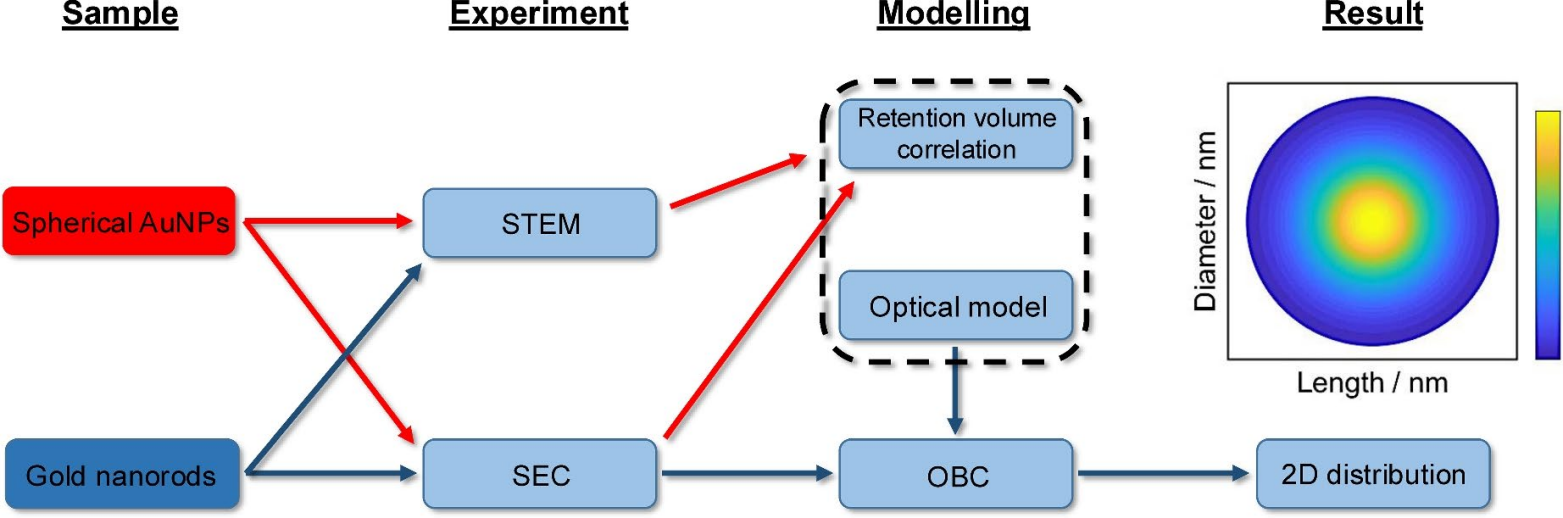
Workflow for the 2D characterization of AuNRs by combination of SEC and OBC. Red arrows indicate the characterization of spherical AuNPs by STEM and SEC and their application for retention volume correlation. Blue arrows indicate the characterization of AuNRs by STEM and SEC and the general procedure of OBC to determine 2D PSDs.

The flow chart depicted in Fig. 2 provides an overview of the data analysis procedure developed in this study. Spherical AuNPs are measured first via STEM and SEC for a determination of the retention volume–size correlation, a prerequisite for the accurate application of the OBC method. The calibration curve must only be constructed once and is applicable for various types of particles, which have repulsive interactions with the stationary phase material^34–37,39^. Next, AuNRs are measured via SEC, which provides the chromatograms and associated extinction spectra for each retention volume. A baseline correction is further applied to the chromatogram by means of a linear regression to account for intensity drifts of the lamp during the SEC measurement. For the OBC analysis, a predetermined number of intervals is created (here 100 intervals) for the normalized cumulative distribution of the retention volume at 480 nm, each of which represents an identical increment in extinction. 480 nm is selected because there is no particular plasmon resonance feature at this wavelength as illustrated in Fig. 4a and in Fig. S1 in the Supporting Information.

Each interval is then determined by its mean retention volume and the full extinction spectrum for all measured wavelengths (200–800 nm). Next, a linearly spaced array with aspect ratios from 1.2 to 4 (29 grid points), which is independent of the retention volume interval, is initiated in order to derive the optical spectra depending on the diameters and lengths of the AuNRs (for details see next section). For each interval, the mean retention volume is converted to a core diameter using the correlation function obtained from the STEM-SEC measurements of the spherical AuNPs. The core diameter values can be converted to hydrodynamic sphere diameters, when making assumptions on the thickness of the ligand shell and solvation. For each hydrodynamic diameter, all possible 29 length-diameter combinations are then calculated based on the given aspect ratios and the shape-dependent translational friction coefficient estimated by Hansen^40^. Using a non-negative linear least-square solver, the number concentrations of the different length-diameter combinations (aspect ratios) are determined for each interval. To generate the full 2D number distribution, the number concentrations from all intervals/spectra are finally put together and categorized based on their diameters and lengths. Further information about the mathematical background behind the OBC method can be found in the original work^21^.

#### Optical modeling

In line with the original implementation of the OBC method for the 2D characterization of AuNRs by multiwavelength AUC, only species with a theoretical peak maximum for the longitudinal surface plasmon resonance mode between the selected maximum wavelength of 800 nm (limit given by the DAD) and the local spectral minimum at around 550 nm are taken into consideration^21^. As the transverse mode’s resonant peak is less susceptible to shape anisotropy, it is not further considered for the OBC analysis. To predict the optical characteristics of plasmonic NPs, we rely on the Gans extension of Mie theory^41,42^, which solely takes into account polarization along the nanorods’ length axis and provides a fast and cost-effective access to the extinction coefficients^43^. While not being as accurate and flexible as other approaches such as finite element modeling, it proved to be sufficient for its application in OBC^21^. However, in order to apply the Gans extension of the Mie theory for the determination of the aspect ratio of AuNR, adjustments of the dielectric constant of the surrounding medium are necessary. Specifically, the refractive index/dielectric constant is adjusted to minimize the deviation between the STEM and OBC derived aspect ratio distributions. The shell of the organic ligand layer is assumed to be optically transparent.

## Results

### Retention volume correlation

**Fig. 3:**
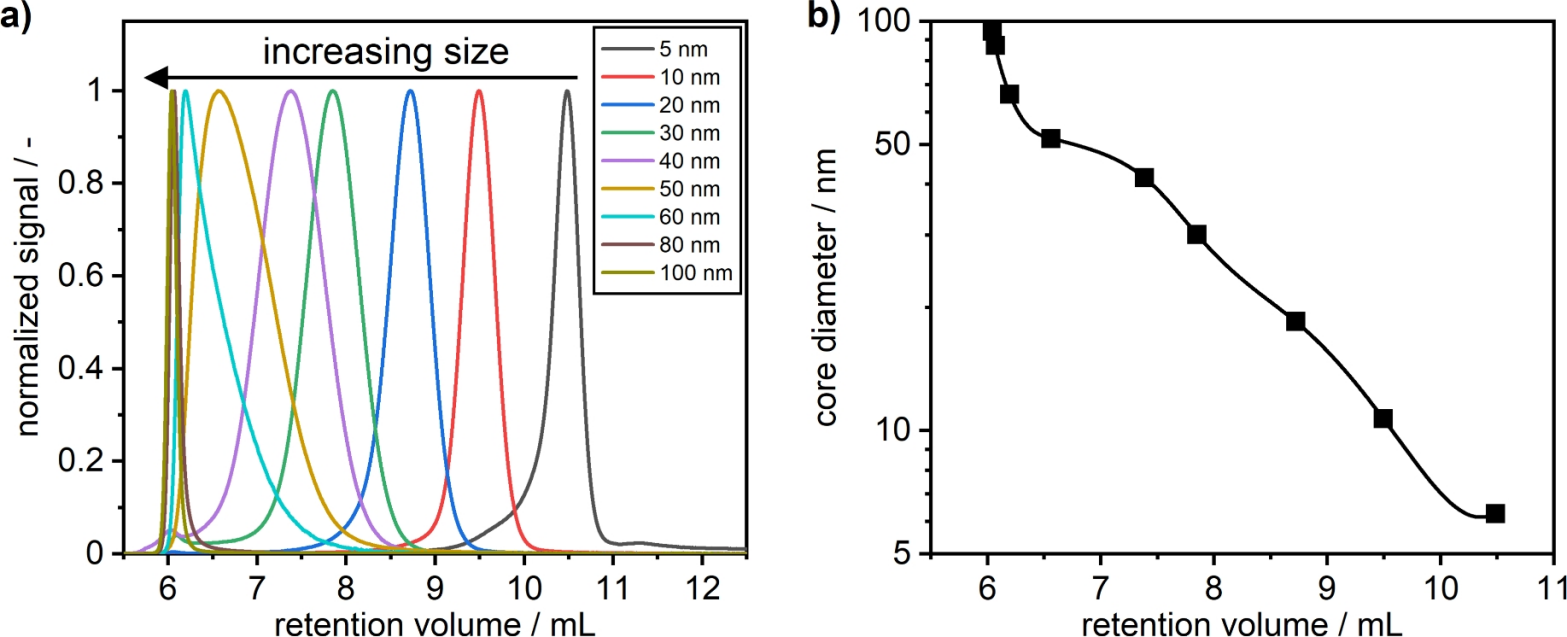
(**a**) Overlaid chromatograms of AuNP standards with diameters of 100, 80, 60, 50, 40, 30, 20, 10 and 5 nm (from left to right) measured at 520 nm. (**b**) Resulting calibration curve determined from STEM core diameters and respective retention volumes of peak maxima.

To retrieve the complete 2D distributions of AuNRs, the diameter distributions of spherical AuNPs were first determined as demonstrated in our previous work^34^. By consecutive injections of differently sized spherical AuNPs (Fig. 3a) a calibration curve was obtained from which the 1D PSDs are determined (Fig. 3b).

Since retention volumes increase with decreasing AuNP diameter, the retention is driven by a size exclusion mechanism. While the chromatograms of AuNP sizes from 5 to 60 nm can be clearly distinguished, the 80 nm and 100 nm AuNPs elute at nearly the same retention volume of 6 ml. Furthermore, the peak widths of both dispersions are significantly narrower which indicates that they are unable to penetrate the pore volume and thus elute close or at the total exclusion limit of the column. This is also reflected in the calibration curve (Fig. 3b) where a steep slope at about 6 ml is observed.

From the resulting calibration curve, 1D size distributions can be determined^34^. Herein, we decided to use STEM as benchmark and for calibration due to its superior accuracy compared to DLS, which is essential for the application of the OBC method. This is necessary as minor errors in particle size have a large impact on the derived 2D distributions due to the set aspect ratios, which are derived from the extinction properties of the samples. Since STEM measurements retrieve the core diameter instead of the hydrodynamic size, which is accessed by SEC, the shell thickness needs to be considered for the correlation. However, as the shell thickness is treated as a parameter for the OBC calculation anyways, and both AuNPs and AuNRs are stabilized by citrate, no additional fitting parameter needs to be accounted for the OBC method.

### 2D length-diameter distribution of gold nanorods

**Fig. 4:**
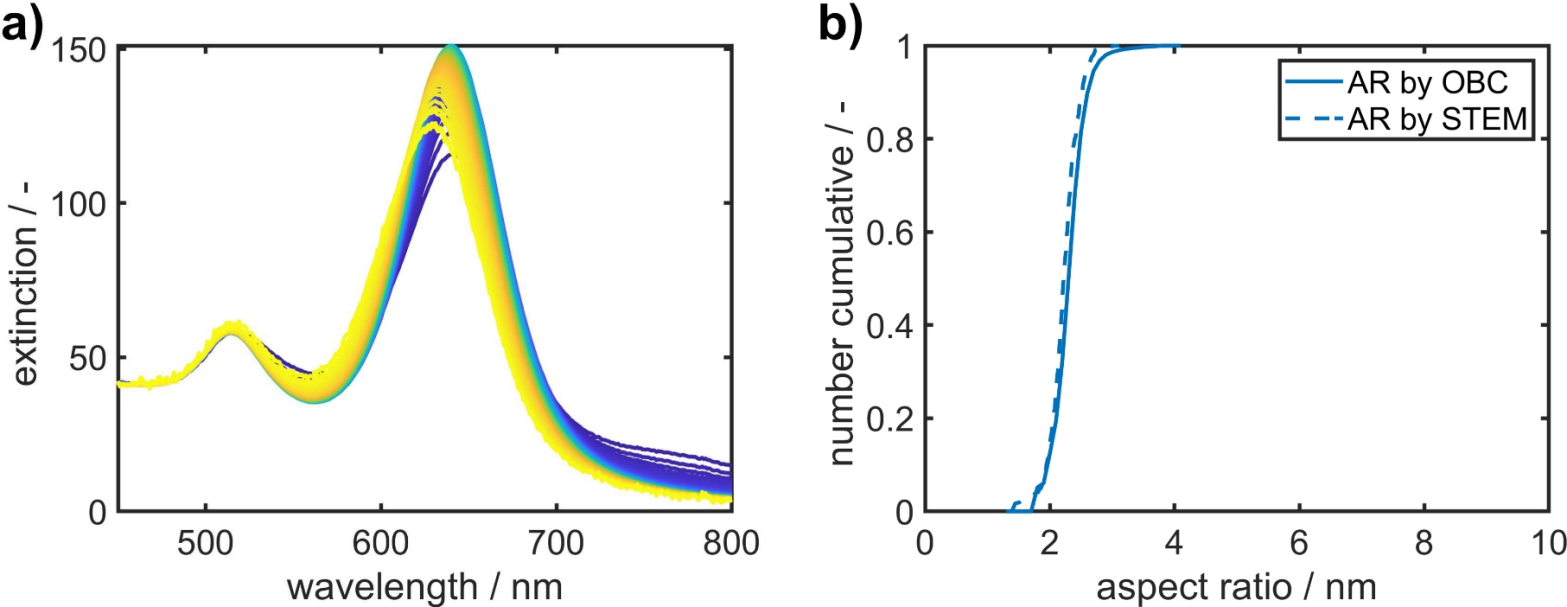
(**a**) Retrieved mean extinction spectra of retention volume intervals for AuNR-650. Blue to yellow color indicates an increase in retention volume and thus a decrease in hydrodynamic diameter. (**b**) Comparison of aspect ratios determined by STEM and OBC using the Gans extension of Mie theory model for AuNR-650.

To verify our OBC methodology for the characterization of 2D distributions of citrate stabilized AuNRs by SEC, we performed the evaluation for AuNRs with four different length and diameter distributions and thus aspect ratios. The samples are denoted according to their longitudinal plasmon peak position as AuNR-600, AuNR-650, AuNR-700 and AuNR-750, respectively. As a reference, we additionally determined the 2D distributions by STEM measurements to validate the OBC results. For the OBC analysis, chromatograms were cut to exclude larger AuNPs and potential agglomerates, which are unable to diffuse into the pores of the stationary phase and thus cannot be separated under these conditions. The modified chromatograms were then evaluated within the 1% to 99% limits of the cumulative extinction weighted distributions. Within these boundaries, the sum chromatograms were divided into 100 equally spaced intervals (weighted by their extinction at 480 nm) from which the mean extinction properties were retrieved within 200 nm and 800 nm as shown in Fig. 4a for AuNR-650. The spectra follow a clear trend of the longitudinal plasmon resonance towards shorter wavelengths with increasing retention volume. The trend is in line with the expected elution order of SEC, since larger AuNPs and more elongated AuNRs elute before smaller ones.

In our previous study, we examined the influence of three optical models (Gans, finite element method (FEM) and longitudinal polarization (LP)) on the 2D analysis results by OBC using AUC^21^. Since the Gans extension provided the best compromise between computational costs and agreement of the 2D distributions determined by AUC and STEM data, when properly adjusting the dielectric constant of the surrounding medium $\epsilon _{m}$ and shell thickness *h*, we applied this model also for the characterization of AuNRs by SEC. In particular, we here used $\epsilon _{m} = 2.09$ and $h = 2.0$ nm for our calculations. The dielectric constant was adjusted in such way that it led to the best agreement in the aspect ratio distributions obtained by TEM and the OBC analysis. Notably, the value of 2.09 is in excellent agreement with the value determined for the AUC-OBC analysis, where a dielectric constant of 2.07 was determined for citrate-stabilized AuNRs. The shell thickness was taken from literature^44,23^. From the mean extinction spectra extracted from the cumulative chromatogram, the mean aspect ratios of the 100 intervals are calculated by the Gans extension (Fig. 4b).

**Fig. 5:**
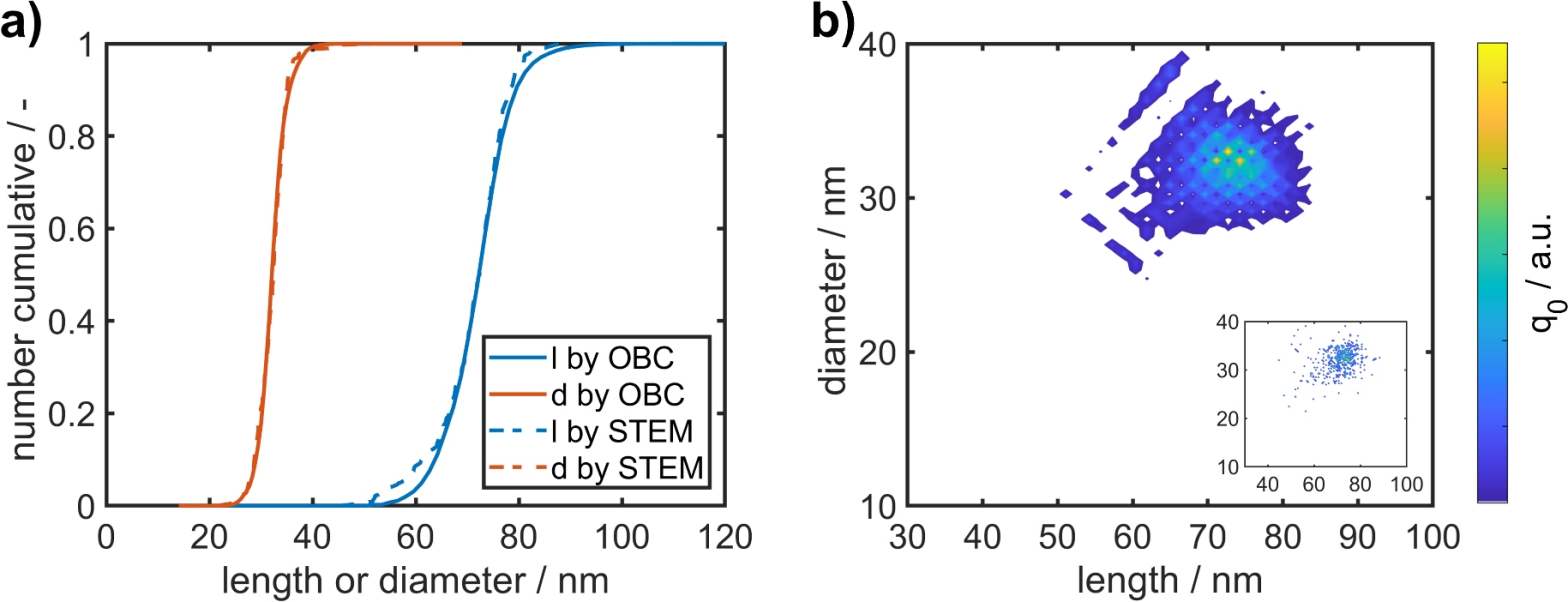
(**a**) Retrieved cumulative length and diameter distributions from STEM and OBC analyses of AuNR-650. (**b**) 2D PSD of AuNR-650 determined by OBC. The inset shows the 2D distribution determined by STEM.

In total, 411 particles of AuNR-650 with aspect ratios > 1.3 were analyzed from STEM images. We neglected AuNRs with smaller aspect ratios, since nearly spherical NPs do not exhibit a pronounced longitudinal plasmon peak which impedes the evaluation by optical methods. The aspect ratio distributions determined by both methods are in very good agreement confirming the quality of the extracted extinction spectra as well as the applicability of the Gans extension to determine the aspect ratio distribution accurately. With the aspect ratios at hand, the length and diameter distributions (Fig. 5a) are calculated by combining the aspect ratio distribution of each interval with the respective particle sizes determined from the SEC calibration curve (Fig. 3b). Again, the distributions determined by STEM and OBC match very well. Since the length and diameter distributions of all intervals are known, the full 2D PSD can be constructed as shown in Fig. 5b.

**Fig. 6:**
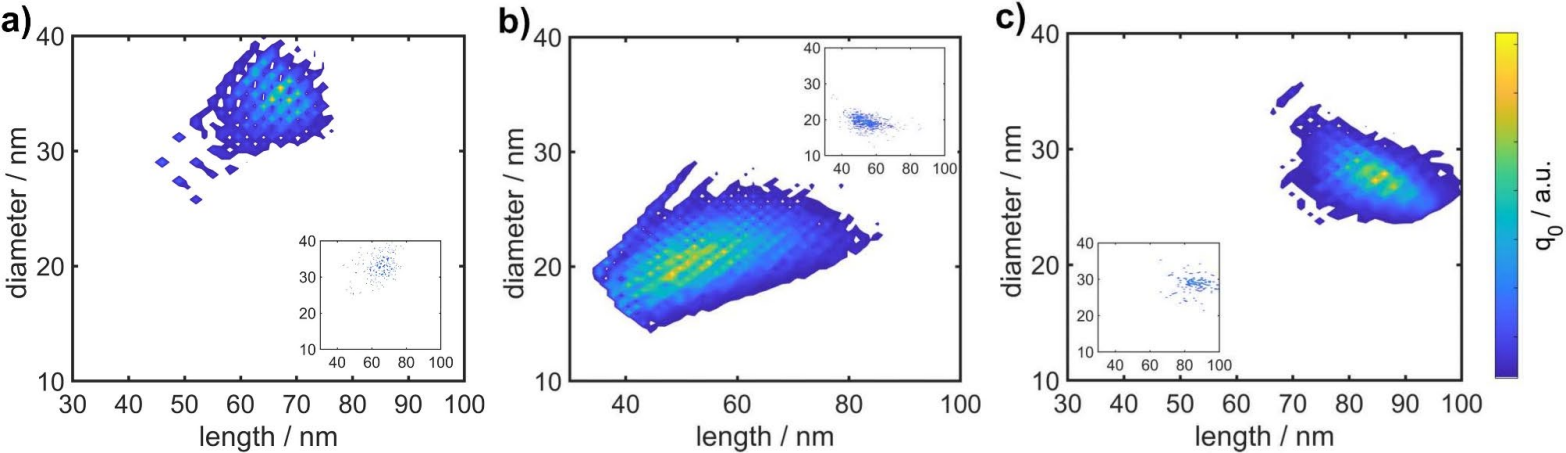
2D PSDs of AuNR-600 (**a**), AuNR-700 (**b**) and AuNR-750 (**c**) determined by OBC. The insets show the respective distributions determined by STEM.

To further validate our methodology, we performed the analysis by OBC and STEM for three more AuNR dispersions. For the STEM characterization, 256 particles of AuNR-600, 521 of AuNR-700 and 155 of AuNR-750 with aspect ratios > 1.3 were evaluated. The resulting 2D PSDs are shown in Fig. 6. As for the previously discussed sample, the 2D distributions determined by OBC and STEM match very well demonstrating that our methodology can be readily applied to a broad range of length, diameter and aspect ratio distributions. Interestingly, the 2D distributions of AuNR-600, AuNR-650 and AuNR-700 show an average diameter that increases with increasing length (their distributions are pointing ‘upwards’ in the graph), whereas the 2D distribution of AuNR-750 shows a decreasing trend in diameter for increasing length (the distribution is oriented ‘downwards’ in the graph). This is an important hint that the conditions during synthesis must be different, an important observation that was so far inaccessible and which can be explained as follows. A 2D distribution is characterized by the mean values of the two variables length (*l*) and diameter (*d*), their variances and covariance. The covariance $\sigma _{ld}$ indicates the relationship between variables *l* and *d*. If $\sigma _{ld}>0$ (pointing ‘upwards’ in the graph), then an increase in *l* is correlated with an increase in *d*; if $\sigma _{ld}<0$ pointing ‘downwards’ in the graph), then an increase in *l* is correlated with a decrease in *d*; if $\sigma _{ld}=0$, then the values of *l* and *d* are uncorrelated, i.e., the distribution is symmetrical^45^. The retrieved extinction spectra and marginalized distributions of aspect ratio and diameter can be found in the supporting information, Figs. S1 to S3.

Despite these promising results, we see some limitations and room for further improvement of our approach in the future, which will be discussed in the following section. As seen for the aspect ratio distributions, a very good agreement between the OBC-SEC and STEM data is generally obtained, confirming the suitability of the optical model used for the OBC. Most importantly, the observed deviations are not one-sided, which would occur due to insufficient calibration by the refractive index of the surrounding medium, and could therefore be attributed to variations in the optical properties of the different AuNRs or to inaccuracies in the used refractive indices of the AuNRs. A current limitation of our approach, considering the modelling of the system’s optical properties, is its inability to resolve particles with low aspect ratios < 2, as the transverse plasmon mode is neglected in the OBC analysis due to the inherent inability to retrieve it from the Gans extension of Mie theory. We are currently working on a more general description of the optical properties of plasmonic nanoparticles in the OBC framework using the T-Matrix method, which is not limited to AuNRs but can also be applied to other materials and geometries. This will allow us to obtain information about anisotropic and spherical particles simultaneously using the OBC analysis as both transverse and longitudinal modes would be correctly accounted for. Another limitation of our current setup is the DAD, which is restricted to a maximum detection wavelength of 800 nm. As a consequence, analysis of AuNRs with aspect ratios > 4 is not possible because the longitudinal plasmon mode is either cut off or not detected at all. Hence, we recommend detection of the samples’ extinction up to a wavelength of 1100 nm, although we could not find any commercial solutions for this at the moment.

Beyond the description of the optical properties and thus the determined aspect ratio distributions, our comparisons of the PSDs obtained by OBC and STEM analysis revealed some deviations in the length and diameter of the AuNRs. On the one hand, this can be explained for STEM by systematic errors during sample deposition prior to analysis and limited statistics in particle counting. On the other hand, the accuracy of the SEC-derived hydrodynamic diameters depends on the data used to calibrate the retention volume-size correlation. Although we used STEM data because of its superior accuracy compared to DLS data, inaccuracies in the correlation cannot be excluded because an interpolation is used to describe the data. This can be seen for the correlation depicted in Fig. 3, where the description of the correlation function for the complete range of particle sizes is of limited accuracy due to a finite number of quadrature points used for the spline interpolation. In addition, the number-weighted average sizes obtained from STEM are correlated with the average extinction-weighted data from SEC. To enhance the precision of the analysis, it would be beneficial to consider not only the mean sizes of the particle standards, but also the complete, ideally overlapping, distributions, which would provide a more comprehensive representation of the system and the correlation. Ideally, these benchmark distributions are not obtained from STEM but AUC, which offers superior resolution and statistics. In this way, a better correlation can be obtained since the OBC analysis does not have to rely on a polynomial fit or interpolation based on a few ($\sim$ 10) data points for calibration.

## Conclusions

In this paper we demonstrated that SEC in combination with OBC is a powerful technique for the 2D characterization of non-spherical nanoparticles within one experiment. By combining hydrodynamic properties with the optical features simultaneously detected during the chromatographic separation, multidimensional properties including length, diameter, aspect ratio and finally entire 2D distributions of AuNRs can be easily and quickly determined with equipment available in most laboratories. Our approach is not only limited to AuNRs but can be transferred to other nanoparticulate materials and geometries such as silver or alloy NPs and bipyramids or triangles as long as optical models for OBC are available. With our current setup, NPs with extinction properties in the visible range can be characterized. By improving the calibration of the SEC and when using column materials with larger pores and advanced detectors, the 2D analysis by OBC-SEC can be applied also to larger NP systems and to the detection in the NIR/IR range offering an even broader field of applications. Moreover, our methodology can be certainly transferred to other surface chemistries as long as repulsive forces dominate the overall interactions between the AuNRs and the stationary phase material. For instance, CTAB-stabilized AuNRs bearing positive zeta potentials should be separated on positively charged stationary phases. In this study, we used commercially available AuNRs dispersed in well-defined solvents. However, potential impurities or buffers from the synthesis procedure would either be separated from the much larger AuNRs directly at the head of the column or do not contribute to the extinction in the relevant wavelength range. The fractionation by SEC depends on differences in the hydrodynamic sizes of the particles to be separated. In principle, particles of different shape but similar hydrodynamic size cannot be separated by SEC. This case applies, for instance, to the fractionation of rods and spheres with the same diffusion coefficient, a case which often occurs in practice since the outcome of a NR synthesis often includes spherical NPs as a by-product. In this case, interaction chromatography is a promising option, which is currently under development in our group. Having the full spectrum of NP chromatography methodology at hand, the holistic characterization of non-spherical NPs can be easily applied to better understand so far inaccessible details of the synthesis. This will lay the foundation to high-purity production of size and shape-selected NPs.

## Experimental section and methods

### Materials

All chemicals were used without further treatment and purification. Spherical, citrate-stabilized AuPs of different diameters (5, 10, 20, 30, 40, 50, 60, 80, 100 nm) for the construction of the calibration curve were purchased from nanoComposix (San Diego, California, United States). AuNRs with different aspect ratios (longitudinal plasmon resonances of 600, 650, 700 and 750 nm) were purchased from Nanopartz (Loveland, Colorado, United States). Ammonium acetate (BioUltra, $\ge$ 99%) and sodium dodecyl sulfate (BioUltra, $\ge$ 99%) were from Sigma-Aldrich (St. Louis, Missouri, United States). For all experiments, Millipore water ($18.2\, {\text{M}}\Omega$) was provided by a PURELAB flex water purification system from ELGA LabWater (High Wycombe, UK).

### Preparation of gold nanorods

For chromatographic experiments, 0.9 ml of AuNRs were mixed with 0.1 ml of an aqueous 20 mM SDS solution to reach a final SDS concentration of 2 mM within the dispersion. This concentration is equal to the SDS concentration in the mobile phase.

### Scanning transmission electron microscopy

STEM measurements of AuNPs and AuNRs were performed in a probe-corrected Thermo Fisher Scientific Spectra 200 C-FEG microscope. The microscope was operated in STEM mode using a high-angle annular dark-field (HAADF) detector, a beam current of 120 pA, acceleration voltage of 200 kV and a camera length of 98 mm.

Prior to the measurements, nanoparticles were drop-cast onto a carbon film coated copper TEM grid (200 mesh—Plano GmbH). Size distributions were obtained by manual measurements via a straight-line tool within the software ImageJ (version 1.43u)^46^.

### Size-exclusion chromatography

Chromatographic experiments were performed with an Ultimate 3000 UHPLC setup (Thermo Fisher Scientific Inc., Waltham, MA, USA). The setup included a solvent rack (SR-3000), a quaternary pump (LPG-3400SD), an autosampler (WPS-3000SL), a column thermostat (TCC-3000RS), a diode-array detector (DAD-3000) and a fraction collector (Fraction Collector F). For the separations, a Reprosil Saphir column (Altmann Analytik, Munich, Germany) with 10 $\upmu$m particle size, 100 nm pore size, 300 mm length and 8 mm diameter was used. The aqueous mobile phase of 2 mM SDS and 8 mM ammonium acetate and the temperature of 25 ${\,}^\circ$C was kept constant during all experiments. Column calibration with spherical AuNPs was performed at a flow rate of 0.5 ml/min while AuNRs were analyzed at 0.2 ml/min. To characterize the 2D-distribution of AuNRs by optical back coupling, 3D-chromatograms were recorded within 200 nm and 800 nm using an optical band width of 1 nm and a data collection rate of 5 Hz.

## Supplementary Information

Supplementary Figures.

## Data Availability

The data that support the findings of this study are openly available in Zenodo at 10.5281/zenodo.14679344, reference number 14679344.
