# Supplementary material for: Characterization of the two-dimensional length and diameter distributions of gold nanorods by size exclusion chromatography
Source: Sci Rep. 2025 Mar 12;15:8501. doi: 10.1038/s41598-025-90941-0 (PMC11903794; doi:10.1038/s41598-025-90941-0)
Supplement: Supplementary file 1 — Supplementary Figures. [file 41598_2025_90941_MOESM1_ESM.docx]

**Supporting information**

Characterization of the two-dimensional length and diameter distributions of gold nanorods by size exclusion chromatography

Lukas Hartmann^a,b^, Nabi Traoré^a,b^, Wolfgang Peukert^a,b^, Johannes Walter^a,b,*^

^a^ Institute of Particle Technology (LFG), Friedrich-Alexander-Universität Erlangen-Nürnberg (FAU), Cauerstraße 4, 91058 Erlangen, Germany.

^b^ Interdisciplinary Center for Functional Particle Systems (FPS), Friedrich-Alexander-Universität Erlangen-Nürnberg (FAU), Haberstraße 9a, 91058 Erlangen, Germany

* Corresponding Author

E-Mail: johannes.walter@fau.de

**SI1: Retrieved extinction spectra of AuNR-600, AuNR-700 and AuNR-750**


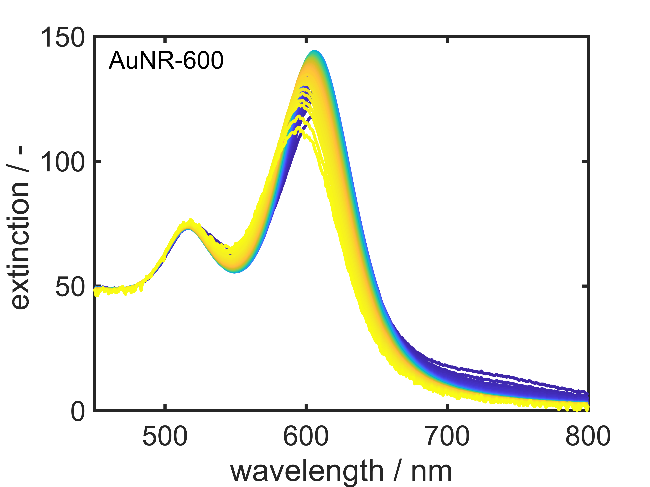

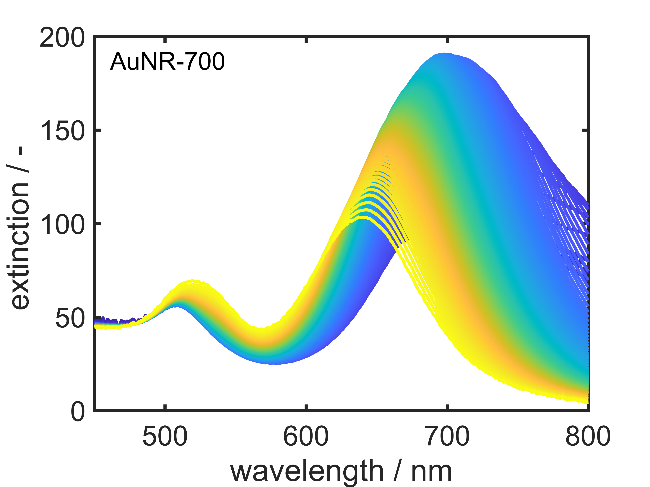


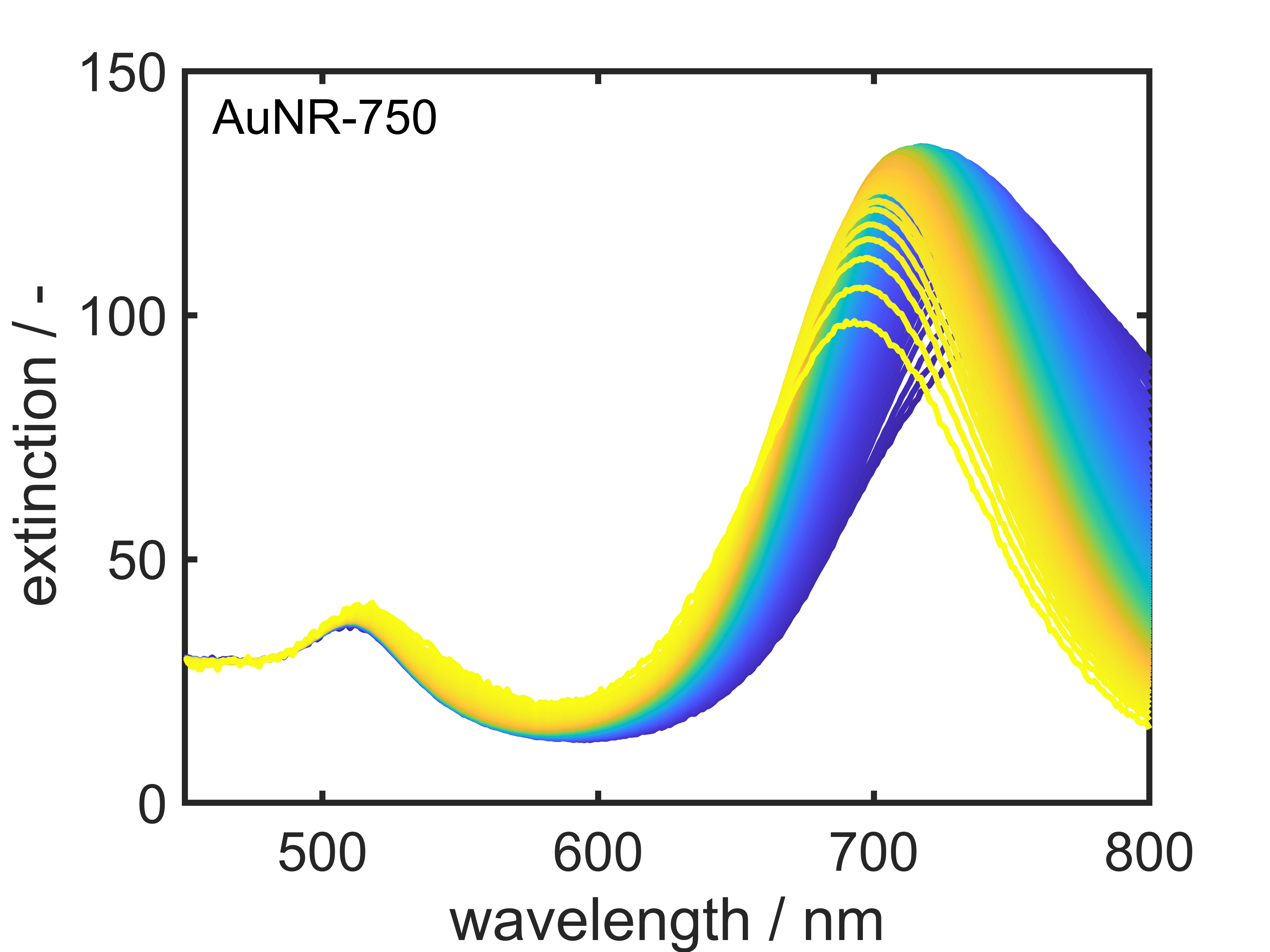


Figure S1: Retrieved extinction spectra of AuNR-600 (top left), AuNR-700 (top right) and AuNR-750 (bottom) for the different retention time intervals. Blue to yellow color indicates an increase in retention time and thus a decrease in hydrodynamic diameter.

**SI2: Aspect ratio distributions of AuNR-600, AuNR-700 and AuNR-750**


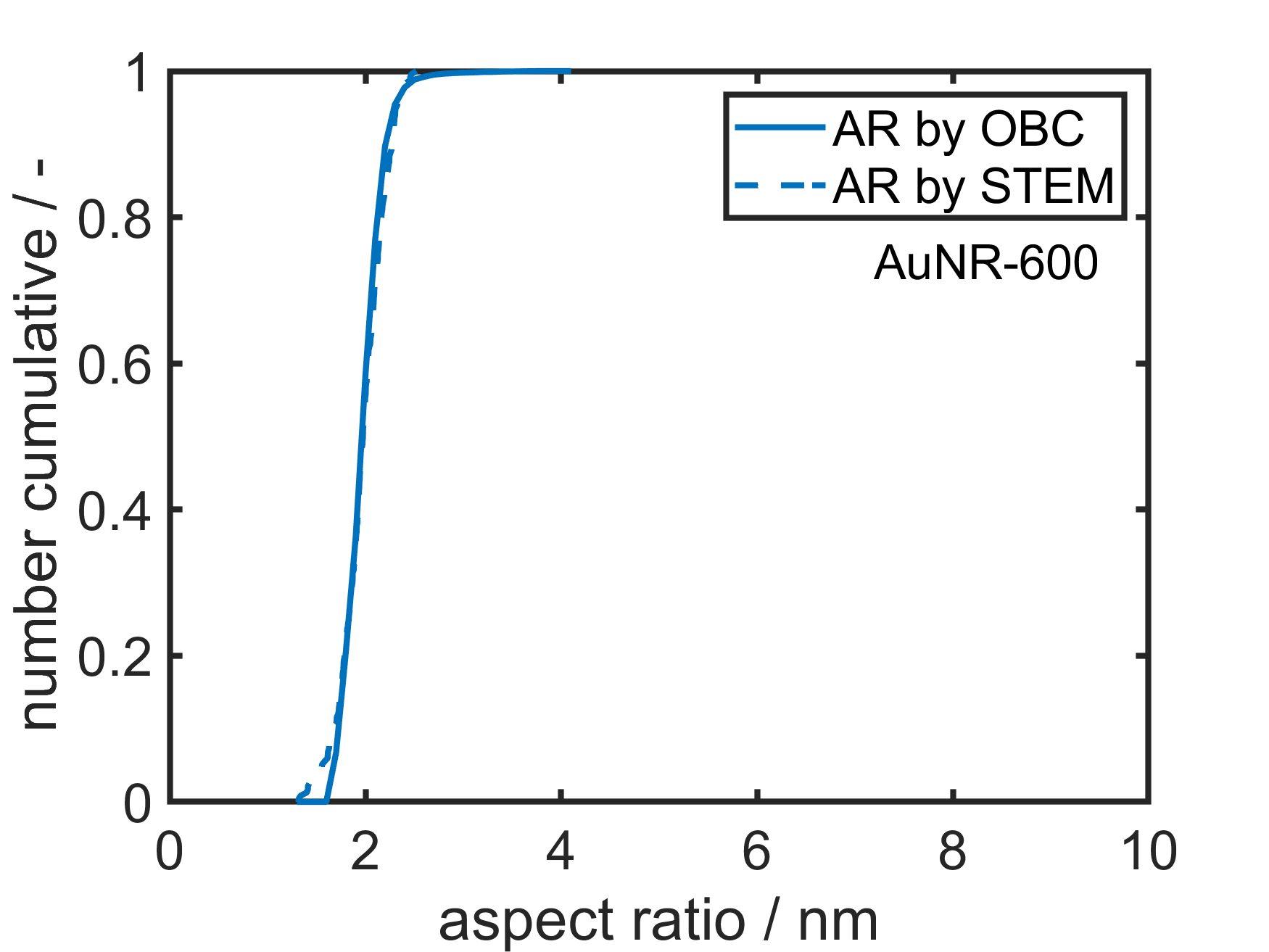

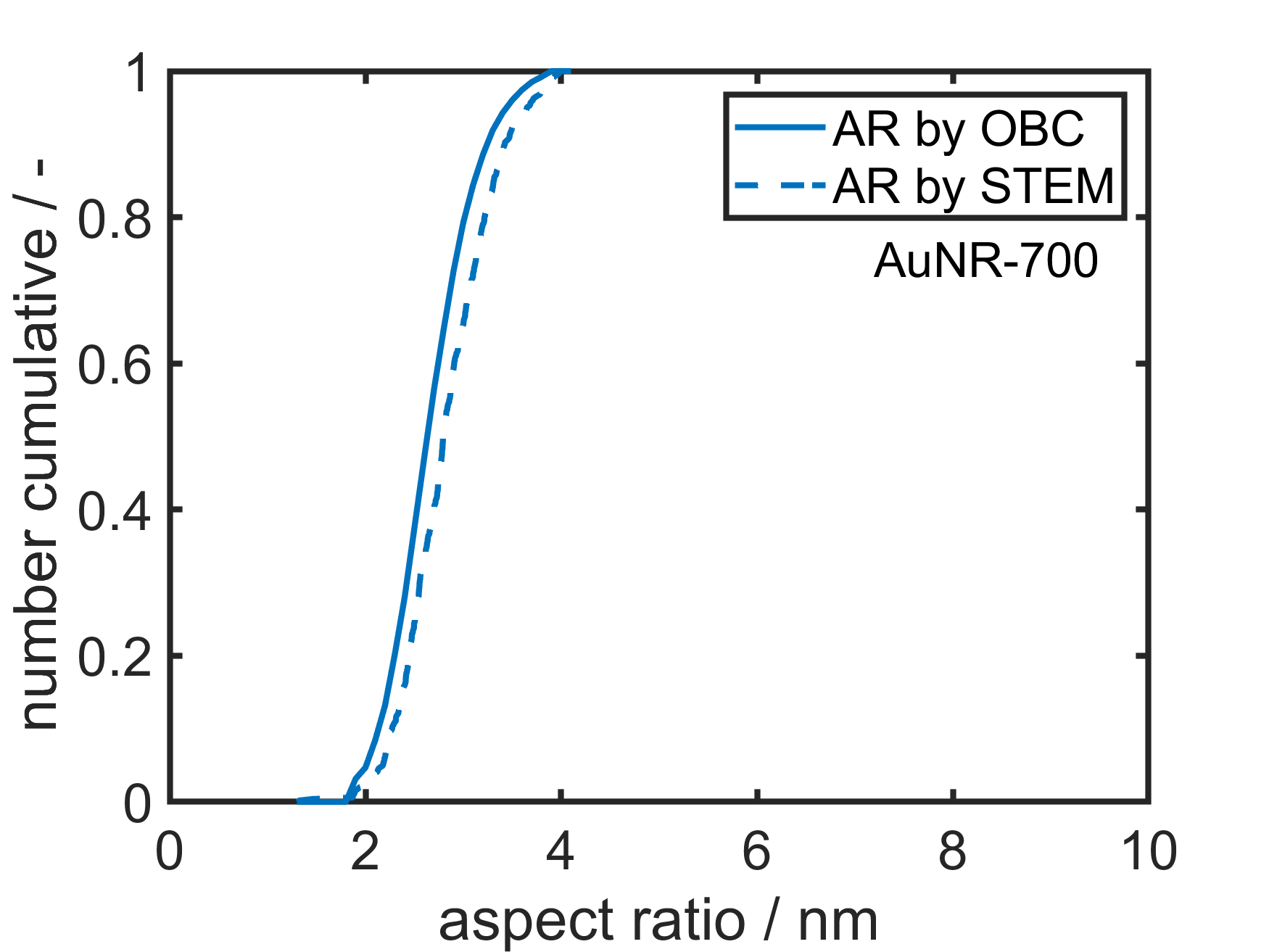

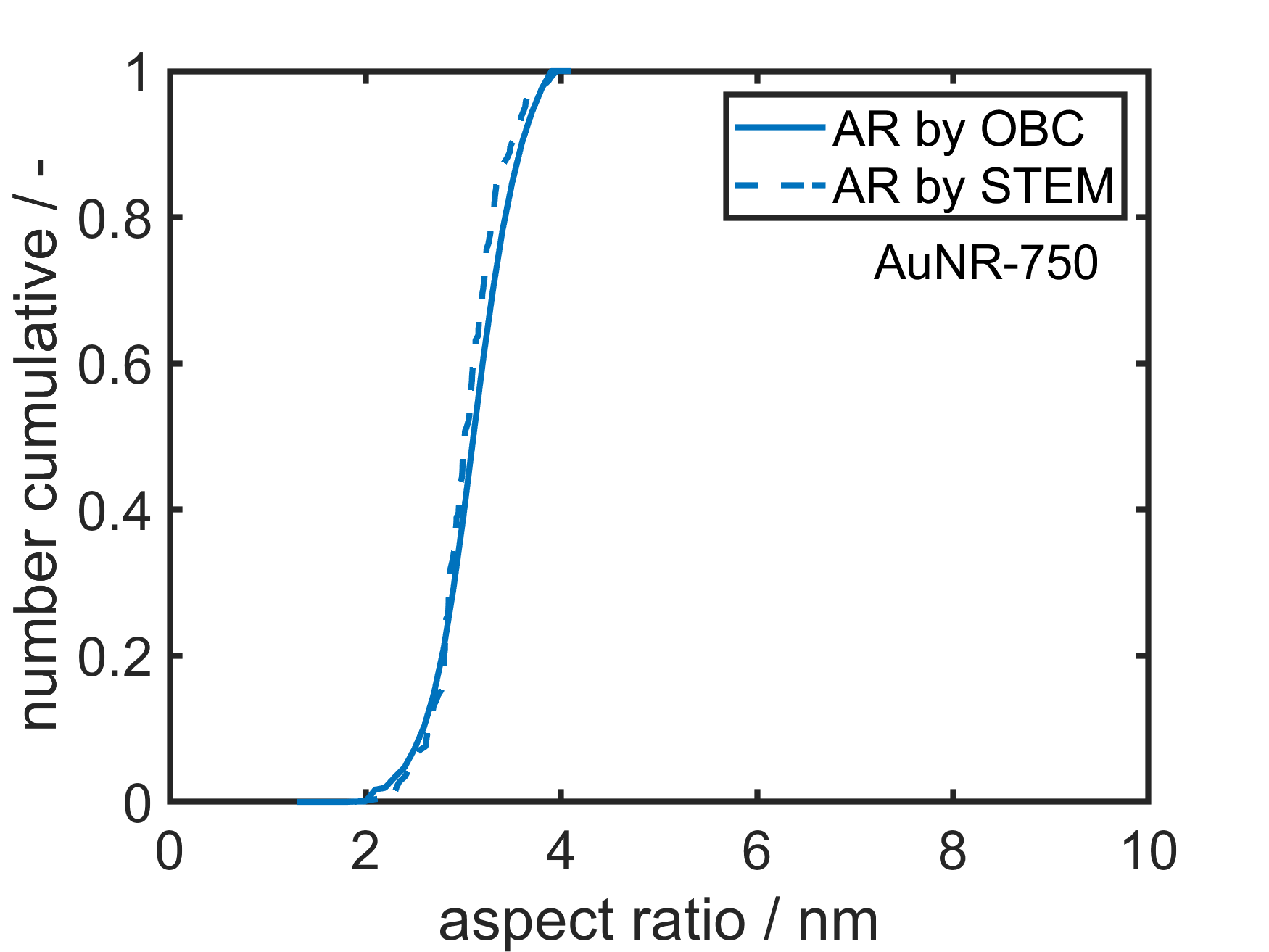


Figure S2: Cumulative aspect ratio distributions of AuNR-600 (top left), AuNR-700 (top right) and AuNR-750 (bottom) as retrieved by the OBC analyses and comparison with STEM data.

# **SI3: Length and diameter distributions of AuNR-600, AuNR-700 and AuNR-750**


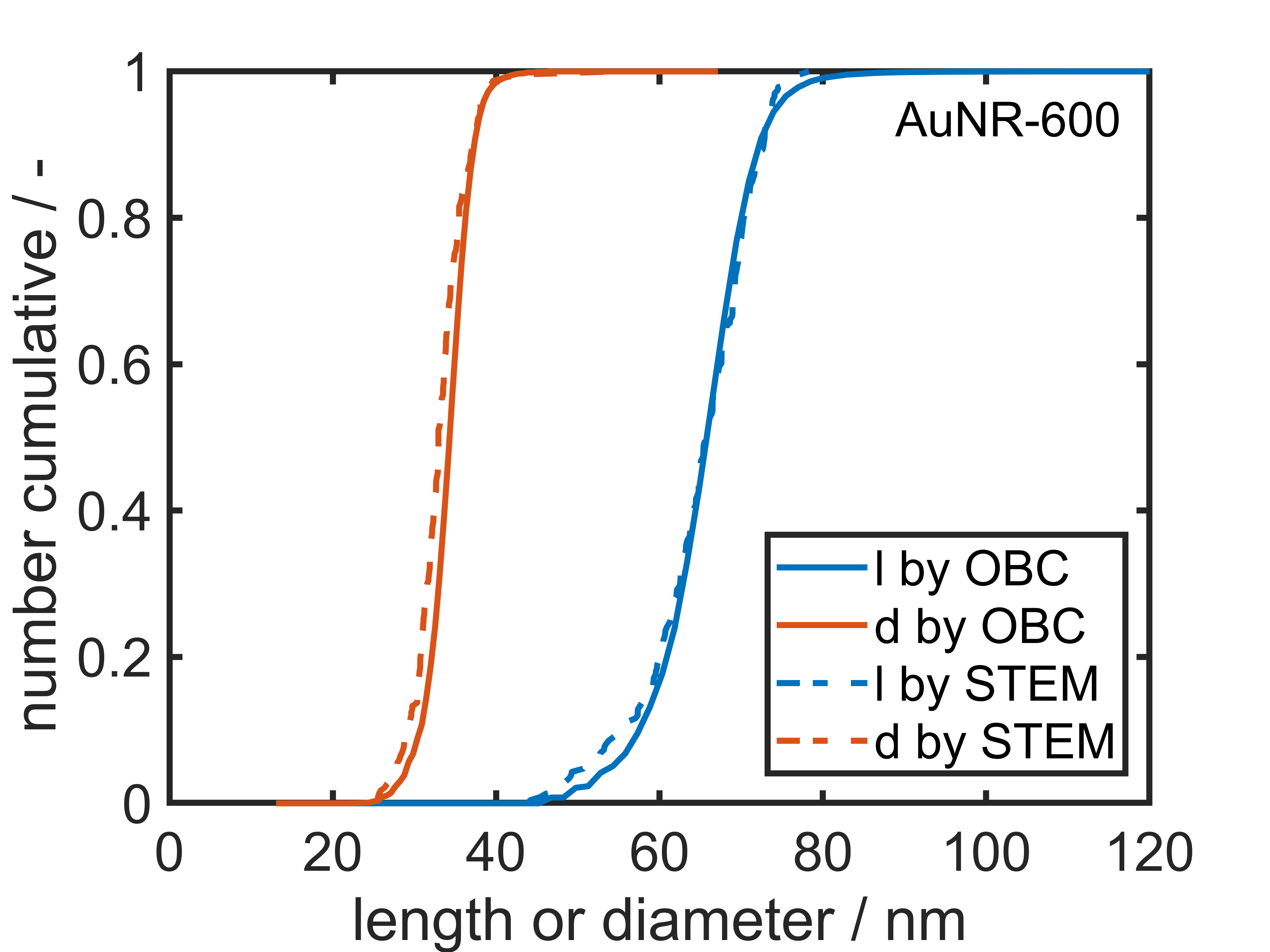

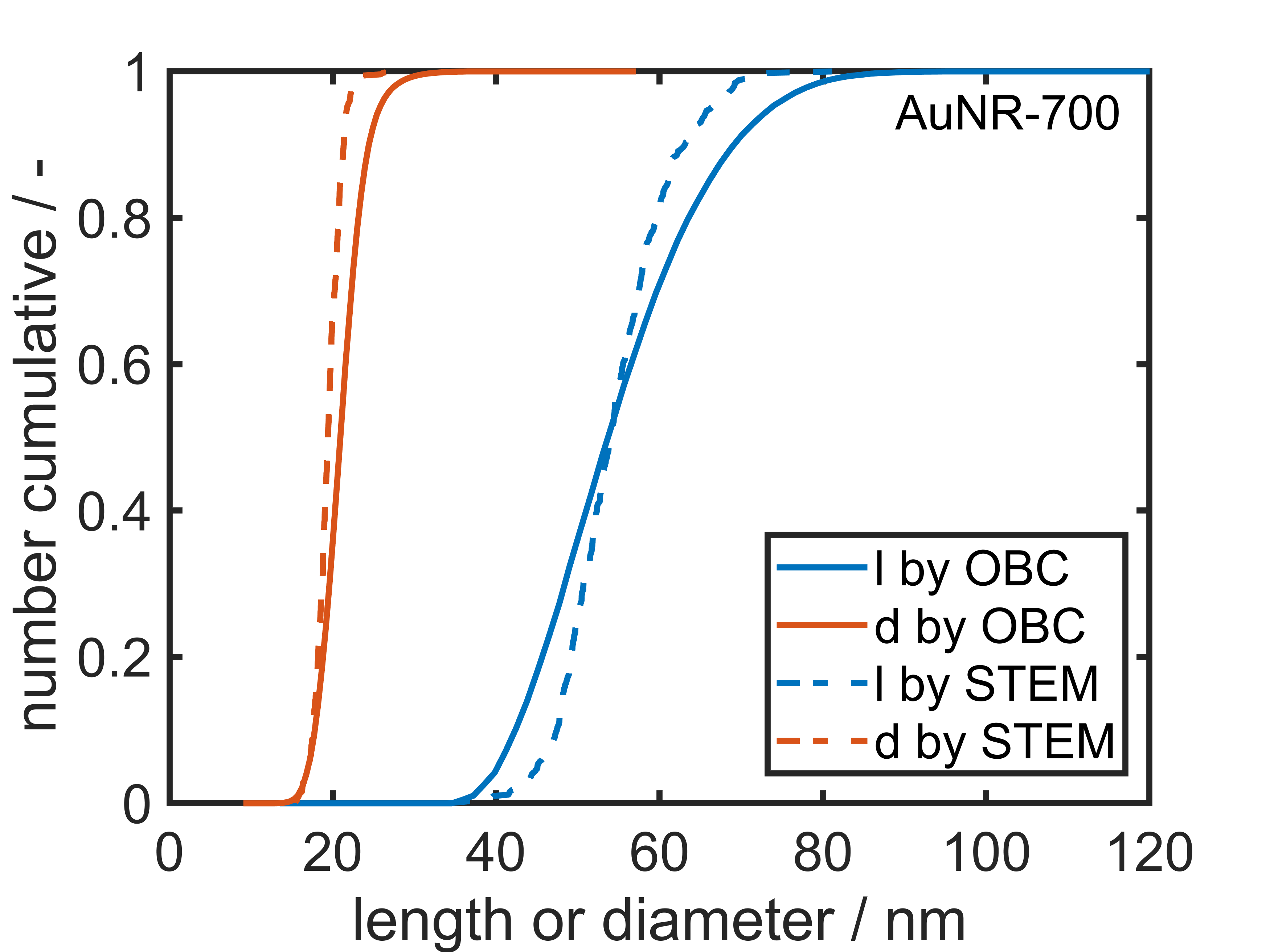

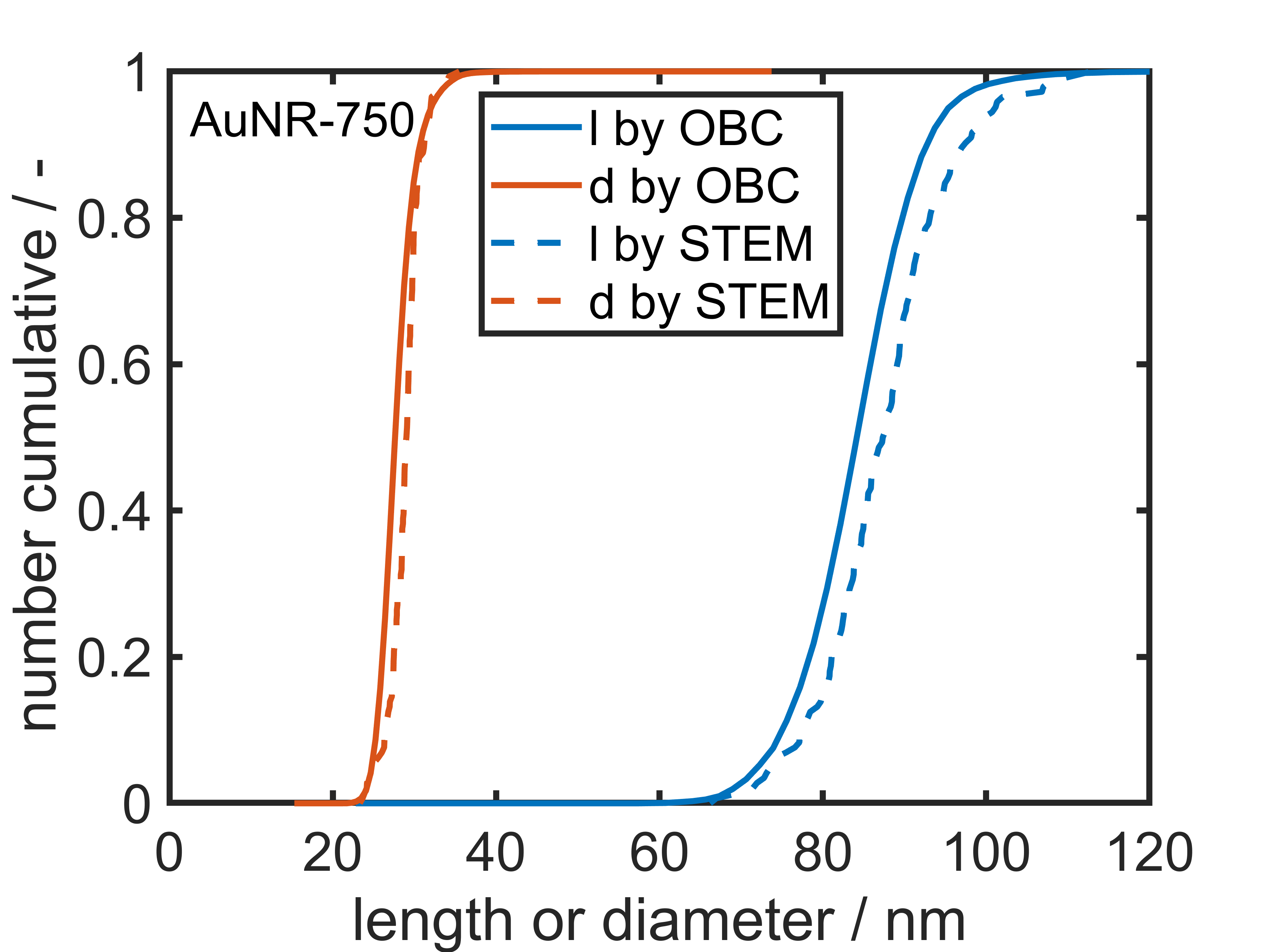


Figure S3: Cumulative length and diameter distributions of AuNR-600 (top left), AuNR-700 (top right) and AuNR-750 (bottom) as retrieved by the OBC analyses and comparison with STEM data.
